# Localized Pulmonary Nocardia farcinica Infection As the Presenting Symptom of Acquired Immunodeficiency Syndrome

**DOI:** 10.7759/cureus.17611

**Published:** 2021-08-31

**Authors:** Nso Nso, Mahmoud Nassar, Laura M Guzman Perez, Tanveer Shaukat, Theo Trandafirescu

**Affiliations:** 1 Internal Medicine, Icahn School of Medicine at Mount Sinai, Queens Hospital Center, New York, USA; 2 Internal Medicine, Icahn School of Medicine at Mount Sinai, Queens Hospital Center, New York , USA

**Keywords:** acquired immune deficiency syndrome (aids), human immunodeficiency virus (hiv), localized, pulmonary, nocardia farcinica, infection

## Abstract

Nocardiosis is an opportunistic infection that most commonly affects immunocompromised patients, with clinical presentations ranging from localized to disseminated disease. In the United States, the reported incidence is approximately 500 to 1,000 cases per year, with an observed male-to-female predominance of 3:1. We present the case of a 37-year-old male with a past medical history of malaria who presented with watery, non-bloody diarrhea for four days associated with a fever for two weeks. The human immunodeficiency virus (HIV) was positive in the emergency room before admission. Computerized tomography (CT) of the chest with contrast revealed an irregular pleural base mass in the right lower lobe with several small air foci. CT of the abdomen and pelvis revealed the right pleural base lung mass to be adherent to the diaphragm, as well as mild splenomegaly. The cluster of differentiation 4 (CD4) count was 9 cells/mm^3^. An acid-fast bacilli (AFB) sputum culture was positive for *Nocardia farcinica*. Trimethoprim-sulfamethoxazole was started for a *Nocardia farcinica* pulmonary infection, in addition to antiretroviral therapy. The patient was strongly encouraged to follow-up at the outpatient department.

## Introduction

*Nocardia* species are Gram-positive aerobic rods usually found worldwide in decaying matter, soil, and water. Of the more than eighty identified species of *Nocardia*, about 25 can potentially cause infection in humans. The *Nocardia asteroides* complex causes more than 50% of the infections in humans and includes the *Nocardia farcinica* species [1-2]. In the United States, the reported incidence of nocardiosis is approximately 500 to 1,000 cases per year, with an observed male-to-female predominance of 3:1. Nocardiosis is an opportunistic infection most commonly affecting immunocompromised hosts, with a clinical presentation ranging from localized to disseminated disease [2-4]. Most *Nocardia* infections occur when an immunocompromised host inhales dust particles containing the bacteria, leading to the most common presentation of the disease, namely, pulmonary nocardiosis [5]. All forms of nocardiosis present with nonspecific symptoms. However, these are usually limited to the affected system. Symptoms of pulmonary nocardiosis include fever, fatigue, anorexia, pleuritic chest pain, cough, hemoptysis, and night sweats [1, 5]. We report a case of pulmonary nocardiosis presenting with gastrointestinal symptoms in a 37-year-old male presumed to be immunocompetent.

## Case presentation

**Figure 1:**
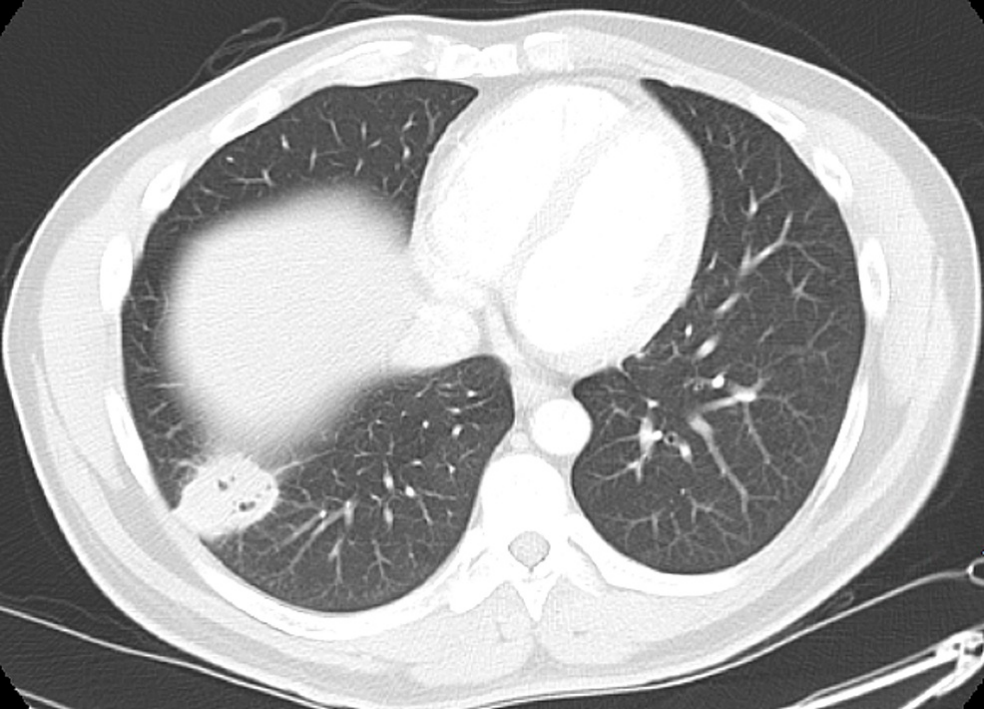
Computerized tomography of the chest with contrast, axial view, shows an irregular, pleural-based mass at the right lower lobe with small air foci.

**Figure 2:**
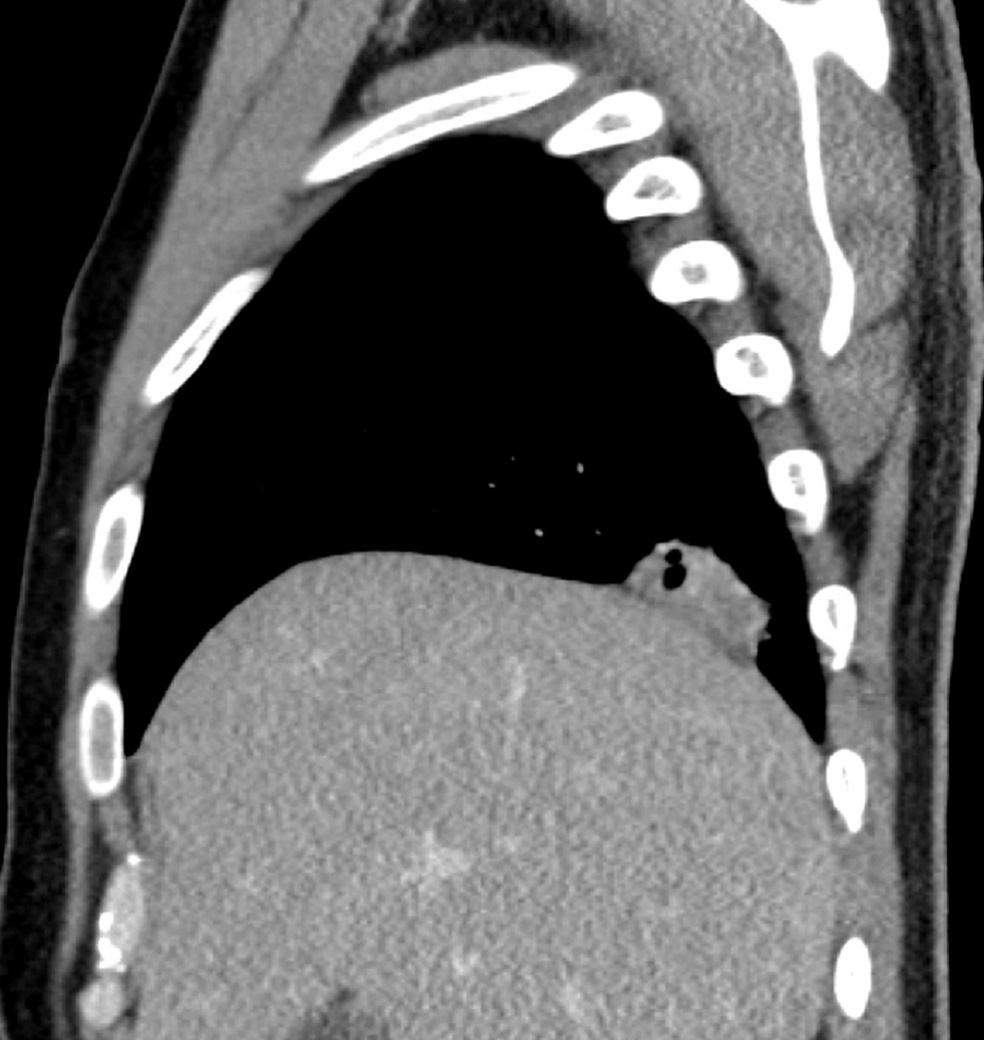
Computerized tomography of the chest with contrast, sagittal view, showed an irregular, pleural-based mass at the right lower lobe with small air foci.

**Figure 3:**
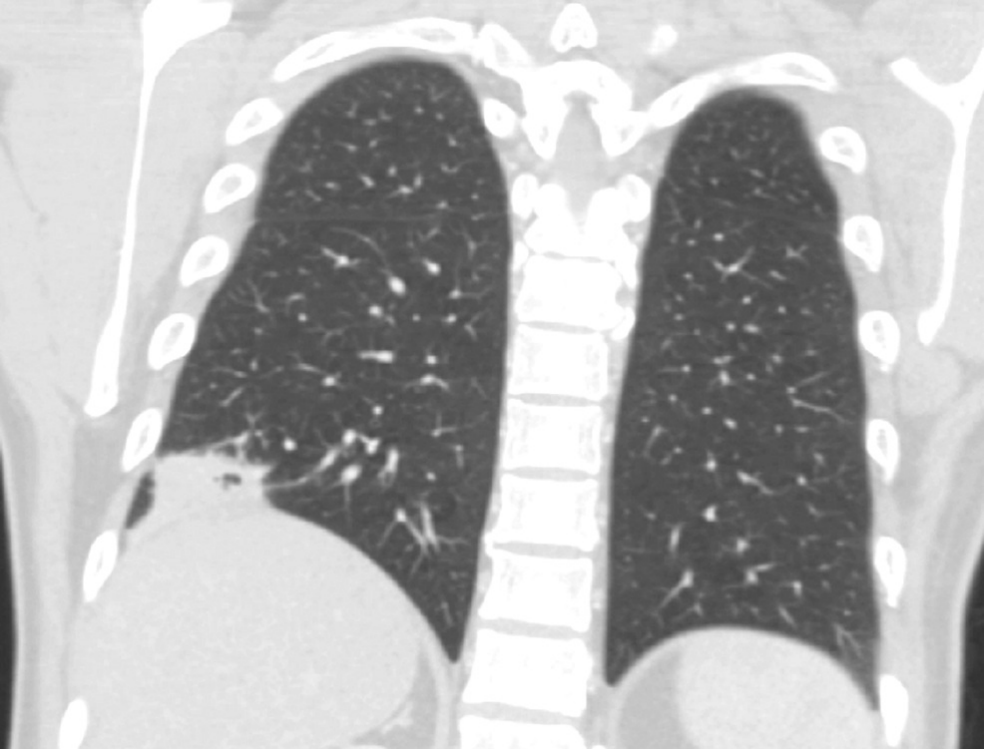
Computerized tomography of the chest with contrast, coronal view, shows an irregular, pleural-based mass at the right lower lobe with small air foci.

A 37-year-old Guyanese male who immigrated to the United States three years ago, with a medical history of malaria, presented to the emergency department (ED) with complaints of watery, non-bloody diarrhea for four days that began abruptly and occurred about three to four times a day, in association with a subjective fever for two weeks. The result of the Abbott human immunodeficiency virus (HIV) Ag/Ab screening test (Abbott Diagnostics, Abbott Park, IL, USA) done in the ED was found to be positive. The patient denied abdominal pain, chills, night sweats, weight loss, recent travel, sick contacts, recent antibiotic use, nausea, vomiting, or changes in diet. The initial vital signs were as follows: temperature 98.3 ͦ F (36.8 ͦ C), blood pressure 116/69 mmHg, pulse 92 beats per minute (bpm), respiratory rate 8/min, and oxygen saturation (SpO_2_) was 98% in room air. The results of the abdominal and rectal exams were unremarkable. The chest x-ray revealed a right lower lung opacity. Computerized tomography (CT) of the chest with contrast demonstrated an irregular, pleural-based mass in the right lower lobe measuring 3.5 x 2.5 cm with several small air foci, likely inflammatory versus infectious in nature (Figures 1-3). A CT scan of the abdomen and pelvis revealed a 4 x 3 cm right pleural-based mass adherent to the diaphragm and mild splenomegaly. The result of the HIV Ab test was positive for HIV-1; the HIV-1 viral load was 55,174, and the absolute CD4 count was 9 cells/mm^3^. The results of the other laboratory tests showed mild neutrophilia (74.35%), mild lymphopenia (11.5%), microcytic anemia (a hemoglobin of 11 g/dL with a mean corpuscular volume of 79.1 fL), and an elevated high-sensitivity C-reactive protein (CRP) (82.4 mg/L). The patient was started on azithromycin, piperacillin-tazobactam, and trimethoprim-sulfamethoxazole. Highly active antiretroviral therapy (HAART) was also started. The acid-fast bacilli (AFB) sputum culture was negative for tuberculosis but returned positive for *Nocardia farcinica*. Trimethoprim-sulfamethoxazole is the leading first-line conventional therapeutic option for *Nocardia farcinica* pulmonary infection and no additional antibiotic was started for this patient. The patient was scheduled to follow-up at the Virology Clinic for the HAART and further management. 

## Discussion

Our newly diagnosed HIV patient with a CD4 count of 9 cells/mm^3^ had subjective fever and diarrhea. The abdominal and pelvic CT scans showed the incidental finding of a right lower lobe cavitary lesion which was ultimately found to be caused by *Nocardia farcinica*. *Nocardia* is a genus of filamentous rod-shaped, aerobic, Gram-positive bacteria which is opportunistic and fastidious and typically causes a localized or systemic suppurative infection in an immunocompromised host [6]. Although the overall incidence of nocardiosis is low, its incidence is higher in patients with HIV compared to the general population [7]. It could be presented as a disseminated disease in HIV patients having a low CD count [8]. Pulmonary nocardiosis often remains undiagnosed due to its nonspecific clinical and radiological presentations, the low diagnostic yield of clinical specimens obtained through noninvasive procedures, the slow growth (up to four weeks) of *Nocardia* on cultures, and/or the very late diagnosis of the condition. This is why it has a high mortality rate [9]. Polymerase chain reaction (PCR) provides a more rapid and accurate diagnosis, but it is available only in a limited number of settings [10]. Earlier studies have highlighted the patterns of lesions ranging from nodules, cavitation, and ground-glass opacities [11]. This case also highlights the pathogenesis of pulmonary nocardiosis, where it begins as a solitary cavitary mass with extension into the pleura and adhesion to the diaphragm that could later spread to other parts of the lungs, causing pleural effusions, empyema, multilobar involvement, and mediastinitis, or it could disseminate hematogenously, commonly to the central nervous system (CNS) [12]. In suspected cases, the differential diagnosis should include bacterial, fungal, and mycobacterial infections in addition to malignancies. Early diagnosis and treatment yield improved clinical outcomes. The *Nocardia asteroides* complex usually responds well to trimethoprim-sulfamethoxazole (TMP-SMX). However, both *Nocardia farcinica* and* Nocardia otitidiscaviarum* can sometimes be resistant to this treatment. Linezolid, amikacin, imipenem, and fluoroquinolones are the options available for *N.* *farcinica* when cultures demonstrate resistance to TMP-SMX [13]. In the case of sulfonamide allergy, either desensitization should be done or the therapy should be guided by the sensitivity results of the culture isolate. The duration of this treatment is three to six months [14].

## Conclusions

A high clinical suspicion is required for the diagnosis of *Nocardia *in immunocompromised patients in whom it is unknown. *Nocardia* could be the initial presentation of an HIV infection. It should be noted that a good medical history, physical examination, and appropriate laboratory testing are essential to rule out other infections and malignancies. A multidisciplinary team is required for dealing with complicated or unclear etiological cases.
